# A survey of veterinary student attitudes concerning whether marijuana could have therapeutic value for animals

**DOI:** 10.1371/journal.pone.0219430

**Published:** 2019-07-08

**Authors:** Nadine A. Vogt, Jan M. Sargeant, Christian P. G. Stevens, Jennifer N. Dunn

**Affiliations:** 1 Department of Population Medicine, Ontario Veterinary College, University of Guelph, Guelph, Ontario, Canada; 2 Centre for Public Health and Zoonoses, University of Guelph, Guelph, Ontario, Canada; 3 Department of Philosophy, King’s College London, Strand, London, England; University of Lincoln, UNITED KINGDOM

## Abstract

Marijuana is increasingly recognized for its therapeutic value in human medicine. Although most veterinary research to date has been concerned with marijuana toxicity, there is some interest in the potential therapeutic value of marijuana in veterinary medicine. With the recent legalization of marijuana for recreational use in Canada in October 2018, there is a need for veterinarians and veterinary students to be in a position to address client questions and concerns on this topic. We distributed a questionnaire to current veterinary students at the Ontario Veterinary College in Guelph, Ontario, to determine their attitude(s) towards marijuana as a potential therapeutic agent in animals. The overall response rate for the questionnaire was 43.5% (207/476). Most students felt that marijuana has potential therapeutic value in animals (53.6%; 111/207), fewer were unsure (38.6%; 80/207), and a small number of students felt that marijuana does not have potential therapeutic value in animals (7.7%; 16/207). Data generated by this questionnaire identified an important distinction between two major active compounds found in marijuana: cannabidiol (CBD) and tetrahydrocannabinol (THC). Potential barriers to use in veterinary practice were also identified, including stigma and toxicity. Finally, many respondents showed an awareness of the limited scientific research regarding the safety and efficacy of marijuana in animals. Until a body of scientific literature on marijuana in animals becomes available, veterinarians may benefit from having an awareness of the different physiological and pharmacokinetic effects produced by different strains (including any adverse effects, and half-life), and a general understanding of current therapeutic applications of marijuana in humans.

## Introduction

*Cannabis sativa* has been cultivated by humans for various religious, industrial, recreational, and medicinal purposes throughout history. Some cultivars of cannabis are predominantly bred for use as a fibre, and are referred to as hemp. Owing to its nutritional value, durability, and strength, hemp frequently appears in the food, textile, and construction industries [1]. *C*. *sativa* is probably best known, however, for its ability to produce a “high”; tetrahydrocannabinol (THC) is the chemical compound responsible for producing this psychoactive effect. Cannabis cultivars which produce a “high” are commonly referred to as marijuana. The classification of a cannabis cultivar as either hemp or marijuana is determined by the concentration of THC, with hemp cultivars containing no more than 0.2 or 0.3% THC by dry weight (in European and North American countries) [1]. Cannabidiol (CBD) is another major chemical compound found in *C*. *sativa*. Unlike THC, CBD does not produce a “high”, and is generally considered non-psychotropic, despite the fact that it has antipsychotic, anxiolytic, and antidepressant effects [2]. Although marijuana cultivars have been of primary interest for therapeutic or medicinal purposes, CBD derived from hemp has also been used for medicinal purposes [1].

THC and CBD are compounds known as cannabinoids. In addition to these dominant cannabinoids, all cannabis cultivars contain hundreds of other cannabinoids which are able to bind to endogenous receptors in the endocannabinoid system of the mammalian central and peripheral nervous systems [3]. As a result, cannabinoids, *inter alia*, may influence cognitive and physiological processes such as mood, appetite, pain-sensation, and memory [3]. In recent years, marijuana has been proposed as an alternative medical treatment for a number of clinical ailments in humans. There is currently peer-reviewed evidence in the human literature to support the use of marijuana as an antiemetic, anticonvulsant, anti-inflammatory, anti-anxiety, anti-cancer agent, as well as for chronic intractable pain, among other indications [4–6]. By contrast, research investigating the potential therapeutic value of marijuana in animals is still in its infancy, with most studies in this field focusing on acquiring basic pharmacokinetic data [7, 8, 9].

The recent legalization of marijuana in Canada ushers in a significant societal change, with Canada being only the second country in the world to legalize marijuana for general use. Although veterinarians in Canada may legally prescribe medical marijuana under the Controlled Drugs and Substances Act, there are currently no products available for animals that have been approved by Health Canada [10]. With the recent change in the legal status of marijuana, there has been an increase in public discourse regarding the potential therapeutic and general health benefits of cannabis products. It thus seems reasonable to anticipate an increase in interest from the public regarding the potential therapeutic applications of marijuana in animals (especially companion animals). Such interest, however, would place veterinarians in an unusual situation; the public has access to a potential therapeutic agent that veterinarians are not in a position to prescribe (due to the absence of animal-approved products). Moreover, the prevailing concern among both veterinarians and researchers regarding marijuana in animals so far has been its role as a toxicant—not as a potential therapeutic agent. Thus, many veterinarians may not feel comfortable or confident discussing marijuana in this new context, which could undermine client confidence and, at worst, result in clients administering cannabis products to their animals without veterinary guidance or oversight.

With the above points in mind, our research objective was to obtain baseline data on the attitudes of veterinarians concerning the use of marijuana as a potential therapeutic agent in animals, as well as the reasons for their beliefs. We distributed a questionnaire to all current veterinary students at the Ontario Veterinary College (Guelph, Ontario) to collect this information from a conveniently accessible population of (prospective) veterinarians. Although our survey population is not necessarily representative of our target population (all practicing veterinarians in Canada), this pilot study was designed as a first step towards understanding how veterinarians are reacting to this rapidly developing field of research and medicine. It is hoped that the data collected and presented here, as well as the brief (critical) discussion to follow of some of the reasons provided by respondents for their views, may prove useful to veterinarians in addressing client questions on this topic. It is also hoped that this research will contribute to a more thorough examination and discussion of this emerging, and potentially controversial, topic in veterinary medicine.

## Materials and methods

A cross-sectional study was performed using an anonymous, online questionnaire to collect information from current Doctor of Veterinary Medicine students (DVM) students at the Ontario Veterinary College (OVC) in Guelph, Ontario, Canada. Prior to administration, the questionnaire was pre-tested by a number of veterinarians and graduate students with training in epidemiology. The questionnaire was administered using Qualtrics Survey Software (Qualtrics, Provo, Utah, USA) and was administered in October and November of 2018. The questionnaire link and survey information were sent to all current DVM students at OVC via class email listservs. Only current DVM students were eligible to participate. Informed consent was obtained at the start of the online questionnaire. The study was reviewed and approved by the Research Ethics Board at the University of Guelph (REB_20180409).

### Questionnaire

To provide baseline demographic data, participants were asked which year of the DVM program they were currently in, as well as their current/proposed stream (i.e., small animal, equine, food animal, rural community practice [formerly mixed]). Information concerning participants’ area(s) of interest in veterinary medicine was also collected to serve as additional demographic data. Participants were asked to indicate their area(s) of interest from among the following options: alternative medicine, welfare/behaviour, surgery, internal medicine, zoo/exotics, public health, research, other (open-ended response). The main question of interest in the questionnaire was as follows:

- Do you feel that medical marijuana could be an effective treatment for some medical conditions in animals? (“Animals” includes companion animals, horses, and farm animals).

Available responses for this question were “Yes”, “No”, and “Unsure”. All participants were asked a follow-up question regarding their reason(s) for their response. If participants answered “Unsure”, they were asked to share in an open-ended question format why they felt unsure. For participants who answered “Yes” and “No”, the follow-up question included several multiple-choice responses, as well as a text box for open-ended responses. Participants were asked to select any and all multiple-choice options that applied to them.

If participants answered “Yes”, the multiple-choice response options were:

- There is scientific evidence that medical marijuana is effective for certain medical conditions in humans.
- There is scientific evidence that medical marijuana is effective for certain medical conditions in animals.
- A veterinarian that you worked for said that it would be effective.
- We discussed it in a class at OVC.

If participants answered “No”, the follow-up question were the following multiple-choice options:

- There is no scientific evidence that medical marijuana is effective in humans.
- There is no scientific evidence that medical marijuana is effective in animals.
- It is too dangerous; the risk of toxicity is too high.
- There are concerns about clients or other client household members using the marijuana themselves.
- Medical marijuana has psychoactive properties which makes it therapeutically undesirable.

The final question in the survey was an open-ended question which screened for any additional concerns or comments.

### Data analysis

Demographic data regarding the distribution of students by year, stream, and veterinary interest(s) were summarized. A summary of responses to the main question of interest was generated, in addition to a summary of responses to the follow-up question regarding justification for the previous response. As our objective was to obtain baseline data on veterinary attitudes, statistical analyses exploring associations between certain characteristics of participants and questionnaire responses were not performed. Moreover, a presentation of results (analytical or descriptive) characterized by stream, year, or veterinary interest would have jeopardized participant anonymity, as there were few responses in certain years and streams. In what follows, we present descriptive statistics aggregated for all years in order to preserve participant anonymity.

#### Analysis of open-ended responses

Open-ended responses were gathered to provide context for quantitative data, and thus were not analyzed using a qualitative research approach. All open-ended responses were open-coded, and codes were created for each response to describe content. We present the most frequently discussed codes, as well as those identified *a priori* to be of particular importance to practicing veterinarians. Illustrative quotes were selected to highlight certain common and otherwise noteworthy concepts; paraphrasing is indicated by the use of square brackets.

## Results

**Fig 1:**
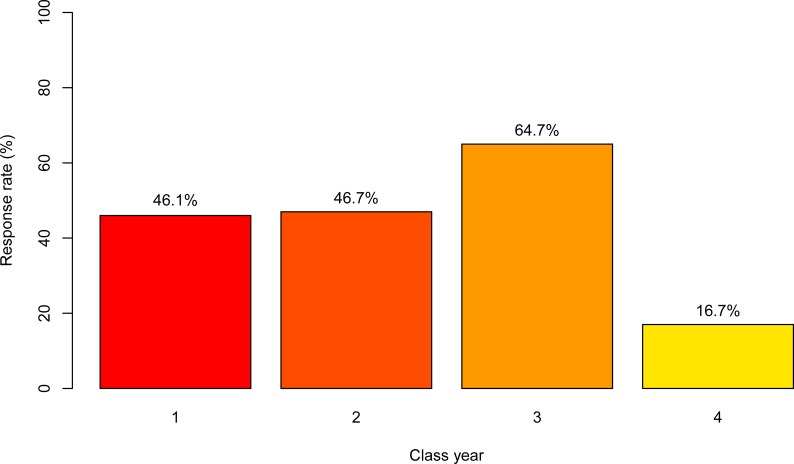
Response rate of veterinary students at the Ontario Veterinary College by class year in a survey of veterinary student attitudes concerning whether marijuana could be an effective treatment for animals.

**Fig 2:**
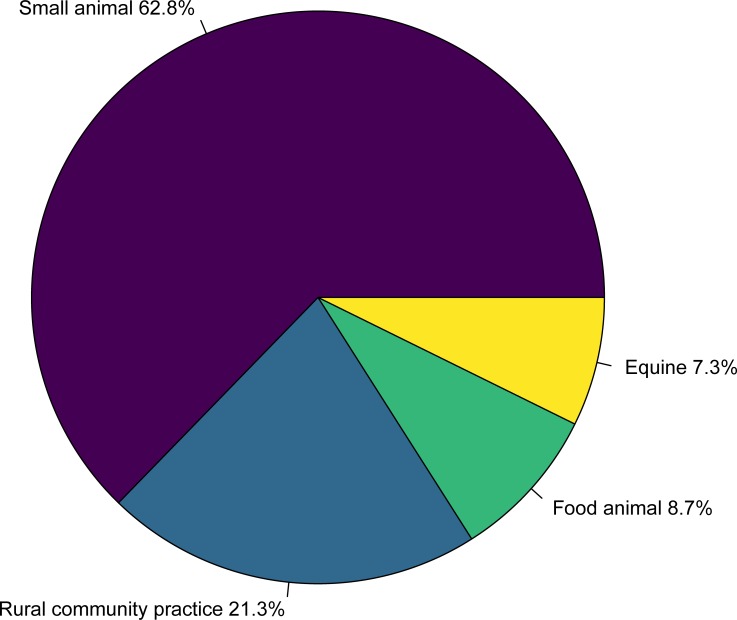
Distribution of veterinary students by stream (or intended stream) at the Ontario Veterinary College among participants in a survey of veterinary student attitudes concerning whether marijuana could be an effective treatment for animals.

**Fig 3:**
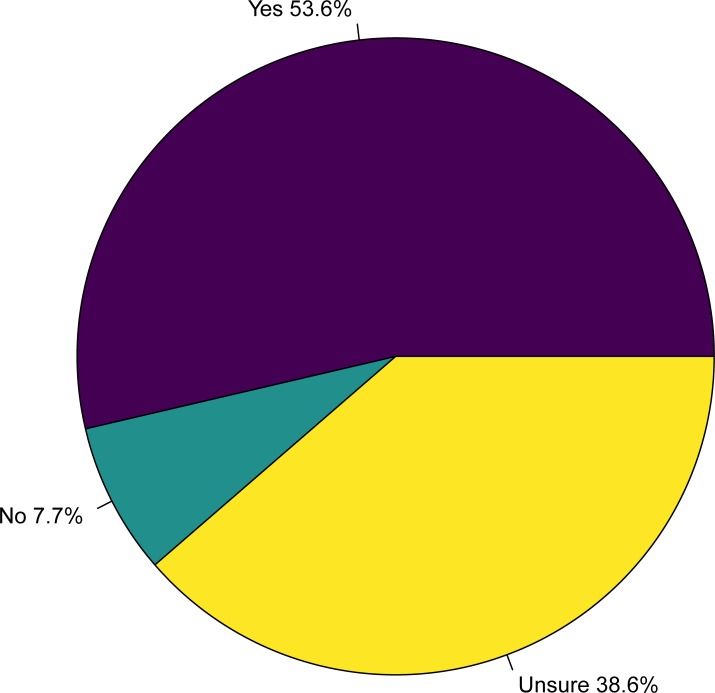
Response to main question of interest “Do you feel that medical marijuana could be an effective treatment for some medical conditions in animals?” among participants in a survey at the Ontario Veterinary College of veterinary student attitudes concerning whether marijuana could be an effective treatment for animals.

A total of 207 responses were received from 476 students, giving an overall response rate of 43.5%. The response rate for each class year was 54/117 in year 1 (46.1%), 56/120 in year 2 (46.7%), 77/119 in year 3 (64.7%), 20/120 in year 4 (16.7%; Fig 1). The distribution of survey respondents by stream was 15/207 equine (7.3%), 18/207 food animal (8.7%), 44/207 rural community practice (21.3%), and 130/207 small animal (62.8%; Fig 2). The number of veterinary interests reported by students ranged between 1 and 7 (mean = 5.6), and a wide variety of interests were reported, with internal medicine (61.8%; 128/207), surgery (66.2%; 137/207), and welfare/behaviour (48.8%; 101/207) reported most commonly. Among respondents, most students (53.6%; 111/207) answered “yes”, that medical marijuana has potential therapeutic value in animals, followed by students who answered “unsure” (38.6%; 80/207), and a small number of students who answered “no” (7.7%; 16/207; Fig 3).

### Reasons for believing marijuana could have potential therapeutic value in animals

Among students who answered “yes”, that medical marijuana could be an effective treatment in animals, the most commonly selected response was that “There is scientific evidence that medical marijuana is effective for certain medical conditions in humans” (90.0%; n = 100/111; Table 1). A smaller number of students indicated that “There is scientific evidence that medical marijuana is effective for certain medical conditions in animals” (14.4%; n = 16/111; Table 1). Five students indicated that “We discussed it in a class at OVC” (4.5%; n = 5/111; Table 1). One student indicated that “A veterinarian that you worked for said that it would be effective” (0.9%; n = 1/111; Table 1). A total of 11 open-text responses were received among students who answered “yes”. Several of these students reported that they were aware of anecdotal evidence supporting the efficacy of marijuana as a treatment in animals (3.6%; n = 4/111). Others stated that marijuana should be considered as a potential treatment option pending further research and scientific evidence to the contrary (4.5%; n = 5/111).

**Table 1:** Reported reasons for response to main question of interest in a survey of veterinary students at the Ontario Veterinary College concerning whether they believe marijuana could be an effective treatment for animals.

Response^a^ to main question of interest: “Do you feel that medical marijuana could be an effective treatment for some medical conditions in animals?”	Reported reasons^b^
“Yes” (n = 111/207)	• There is scientific evidence that medical marijuana is effective for certain medical conditions in **humans** (n = 100) • There is scientific evidence that medical marijuana is effective for certain medical conditions in **animals** (n = 16) • We discussed it in a class at OVC (n = 5) • A veterinarian that you worked for said that it would be effective (n = 1) • Other (n = 11)^c^
“No” (n = 16/207)	• There is no scientific evidence that medical marijuana is effective in **animals** (n = 11) • It is too dangerous, the risk of toxicity is too high (n = 10) • Medical marijuana has psychoactive properties which makes it therapeutically undesirable (n = 7) • There are concerns about clients or other client household members using the marijuana themselves (n = 5) • There is no scientific evidence that medical marijuana is effective in **humans** (n = 4) • Other (n = 1)^c^

^a^Those who responded with “Unsure” (n = 80/207) were requested to provide an open-ended response, analyzed separately and not presented here.

^b^Participants were asked to check any and all applicable responses, therefore totals exceed 100%.

^c^Only multiple-choice reasons are presented here; open-ended text responses indicated by “Other” are presented in text of main article.

Among students who stated they were “unsure”, many students indicated that there is insufficient evidence or research to form a judgement, or that they were unaware of such research or evidence (91.2%; n = 73/80). This concept is demonstrated by the following quote:

“As far as I know, the primary literature for evidence-based medicine is lacking in this field. I am unsure about the risks, side effects, […] drug availability, or [drug] interactions […] that are yet to be thoroughly investigated and documented.”

Other students who stated that they were “unsure” reported concerns regarding the safety of marijuana in animals and potential toxicity (20.0%; n = 16/80).

#### Reasons for believing marijuana does not have potential therapeutic value in animals

Among students who answered “no”, most indicated that “There is no scientific evidence that medical marijuana is effective in animals” (68.8%; n = 11/16). A similar number of students noted that, “It is too dangerous, the risk of toxicity is too high” (62.5%; n = 10/16). Seven students selected the response that, “Medical marijuana has psychoactive properties which makes it therapeutically undesirable” (43.8%; n = 7/16). Fewer students selected: “There are concerns about clients or other client household members using the marijuana themselves” (31.3%; n = 5/16), and “There is no scientific evidence that medical marijuana is effective in humans” (25.0%; n = 4/16). Only one open-text response was received among students who answered “no”; this participant indicated that there is currently no evidence to suggest that medical marijuana is more effective than any of the currently available therapeutic agents.

### Screening for additional concerns

A total of 52 students provided a response to the final open-ended question regarding additional concerns or comments (25.1%; n = 207). These responses highlighted an important distinction, potential barriers to use, and research gaps.

#### Distinction

“There's a difference between THC and CBD effects in animals […] We should […] separate those two out when starting this conversation about marijuana as a medical aid.”

#### Potential barriers

“The stigma around marijuana, regardless of whether it is used recreationally, should not discourage the use of medical marijuana if it shows benefit to animals and humans suffering from certain medical conditions.”

“My main concern about marijuana legalization is that it is a toxicant to small animals, and many patients [end up in emergency clinics] because of accidental ingestion.”

#### Research gaps

“We need […] studies to determine the toxic dose […]”

“More research into the benefits [and] long-term effects [of marijuana in] animals is required [keeping] in mind [that] just because there is evidence in humans does not mean we can transfer it to animals […]”

“I am not against the use of marijuana in veterinary medicine, it's just that there not currently any scientific literature detailing its safety, efficacy, or indications […]”

## Discussion

### Veterinary student attitudes

The majority of veterinary students who participated in the questionnaire either believe that medical marijuana has potential therapeutic value for animals, or are unsure, suspending their judgement pending further evidence. The data produced by this questionnaire suggested a potential misconception concerning the psychoactive properties of marijuana, in addition to several potential barriers to use and research gaps.

### Misconception: All marijuana strains produce a “high”

One student highlighted an important distinction between THC and CBD, noting that they do not have equivalent physiological effects in animals. In humans, it is well known that the proportions each of THC and CBD, as well as the ratio of these cannabinoid compounds determine pharmacological effects [11]. As such, certain strains of marijuana are considered more suitable for certain medical conditions: for example, CBD-dominant strains are preferred for control of epilepsy in children, without a risk of psychoactive effects mediated by THC [12]. Among students who responded that marijuana does not have potential as a therapeutic agent in animals, some were concerned that “medical marijuana has psychoactive properties which makes it therapeutically undesirable.” Agreement with this statement may represent a misconception concerning the pharmacodynamic effects of marijuana: all marijuana strains produce a “high”. Given that some marijuana strains (certain CBD-dominant strains) do not produce a “high” due to a low proportion of THC, the above concern may not apply to such marijuana strains, and is not applicable to hemp strains (which, by definition, contain low proportions of THC).

### Marijuana in veterinary medicine: Potential barriers to use

As highlighted by one participant, the stigma associated with marijuana’s previous legal status as an illicit drug may be a barrier to thinking about marijuana as a therapeutic agent. Furthermore, because marijuana may be toxic for small animals, both clients and veterinarians may be unsure whether marijuana should be considered a potential treatment option for certain conditions, or a toxin. Marijuana toxicity is a problem most commonly associated with dogs, and has been widely described [13]. Following the legalization of cannabis in Colorado, United States, a significant increase in the number of marijuana toxicity cases was observed in two veterinary hospitals [14]. Ingestion of a large dose of marijuana containing THC may produce lethargy, ataxia, vomiting, and seizures in dogs [13]. It is uncertain whether cannabis is directly responsible for fatalities in animals; there is a lack of information regarding lethal dose and the mechanism of fatal toxicity [15]. In the Colorado study, the deaths of two dogs following ingestion of medical grade cannabis edibles containing THC were attributed to cannabis toxicity as a diagnosis of exclusion [14]. Given the rarity of cannabis-related deaths in animals, and lack of basic research on this topic, it may be prudent to suspend judgement on this issue pending further research.

### Research gaps

With marijuana toxicity as the focus of the majority of the veterinary literature on this topic, there is limited information available for veterinarians regarding the efficacy of medical marijuana in animals. Most respondents were aware of this research gap, with some citing anecdotal evidence as the predominant form of support for the efficacy of marijuana in animals. A few studies investigating the efficacy and safety of medical marijuana in dogs have recently become available [7, 8], and further research is likely to follow. Most respondents were also aware of the growing amount of peer-reviewed literature supporting the use of marijuana for certain medical conditions in humans [4–6]. Furthermore, a few students correctly noted that extrapolation from evidence in humans to animals is problematic. Finally, an important research gap was identified regarding the lack of information about toxicity and potential lethality of marijuana in animals.

### Study limitations

Although the response rate for our questionnaire was within the expected range and the demographic data (e.g., distribution of students by stream, varied veterinary interests) consistent with expected distributions, the results may not be representative of the entire veterinary student population at OVC. In particular, the low response rate among fourth year students may reduce our ability to generalize to veterinary students who are close to graduation. The low response rate was likely a result of time constraints for final year students who are participating in clinical rotations, often with unpredictable schedules. The major limitation of our pilot study, however, relates to the generalizability of results from our survey population (veterinary students in Ontario) to our target population (practicing veterinarians in Canada). Future work on this topic involving practicing veterinarians would be useful to potentially validate and build on our findings.

## Conclusion

With the recent legalization of cannabis for humans in Canada, there may be an increase in public interest in the use of medical marijuana as a therapeutic agent in animals. There are also conditions where, if effective, medical marijuana could prove a useful alternative treatment, such as with canine epilepsy, where 20–30% of dogs do not respond to conventional drugs [16]. The results of this questionnaire suggest that veterinary students are interested in acquiring more information on this topic, and that further information may benefit them in confidently addressing client questions and concerns. Participants highlighted certain barriers to the use of medical marijuana in veterinary medicine, including stigma and toxicity. In addition, a potential misconception regarding the variable psychoactive effects of different strains of marijuana was identified: not all strains produce a “high” (e.g., CBD-dominant strains). Aside from the need for further research into the potential therapeutic value of medical marijuana in animals, there is a need for further basic research concerning toxicity and the potential lethality of marijuana in animals. Moving forward, the expertise of prospective and practicing veterinarians in this rapidly developing field may be enhanced through the examination of this topic in veterinary schools and inclusion in continuing education curricula.

## Supporting information

S1 Text Questionnaire. (DOCX)
