# Supplementary material for: A survey of veterinary student attitudes concerning whether marijuana could have therapeutic value for animals
Source: PLoS One. 2019 Jul 8;14(7):e0219430. doi: 10.1371/journal.pone.0219430 (PMC6613771; doi:10.1371/journal.pone.0219430)
Supplement: S1 Text — (DOCX) [file pone.0219430.s001.docx]

Medical marijuana survey

**Background Information**
Veterinarians in Ontario are legally permitted to prescribe medical marijuana under the Controlled Drugs and Substances Act, however, there are currently no available products for animals which have been approved by Health Canada.

Q1 What OVC phase are you in?

- 1
- 2
- 3
- 4

Q2 Which stream are you in/do you plan to select?

- Small animal
- Equine
- Food Animal
- Rural Community Practice (formerly mixed)

Q3 Which of the following areas are of particular interest to you in veterinary medicine? Please check all that apply.

- Alternative Medicine
- Welfare/behaviour
- Surgery
- Internal Medicine
- Zoo/exotics
- Public Health
- Research
- Other: ________________________________________________

Q4 Do you feel that medical marijuana could be an effective treatment for some medical conditions in animals? ("Animals" includes companion animals, horses, and farm animals).

- Yes
- No
- Unsure

*If respondent answered “yes” to Q4:*

Q5 Why do you feel that medical marijuana could be an effective treatment for some medical conditions in animals? Check all that apply.

- There is scientific evidence that medical marijuana is effective for certain medical conditions in **humans**.
- There is scientific evidence that medical marijuana is effective for certain medical conditions in **animals**.
- A veterinarian that you worked for said that it would be effective.
- We discussed it in a class at OVC.
- Other: ________________________________________________
- Prefer not to say.

*If respondent answered “no” to Q4:*

Q5 If you answered no to the previous question, why not? Check all that apply.

- There is no scientific evidence that medical marijuana is effective in **humans**.
- There is no scientific evidence that medical marijuana is effective in **animals**.
- It is too dangerous, the risk of toxicity is too high.
- There are concerns about clients or other client household members using the marijuana themselves.
- Medical marijuana has psychoactive properties which makes it therapeutically undesirable.
- Other: ________________________________________________
- Prefer not to say.

*If respondent answered “unsure” to Q4:*

Q5 Why do you feel unsure about whether medical marijuana could be an effective treatment?

________________________________________________________________

Q6 Do you have any additional comments or thoughts you would like to share?

________________________________________________________________

We thank you for your time spent taking this survey.
Your response has been recorded.
 
If you would like further information about the legal status of medical marijuana for animals in Ontario you may find it at the following link:  
 
https://cvo.org/Utility-Pages/Cannabis-FAQs.aspx
